# Engineered *Mycobacterium smegmatis*-based intrapleural immunotherapy remodels the pleural immune microenvironment for malignant pleural effusion treatment

**DOI:** 10.3389/fphar.2026.1972278

**Published:** 2026-10-26

**Authors:** Leilei Zhou, Zhaoye Qian, Rong Yao, Cheng Chen, Peng Pan, Zhongying Guo, Zhongneng Xu, Yi Mei, Jipeng Wang

**Affiliations:** 1 Department of Oncology, The Affiliated Huai’an No. 1 People’s Hospital of Nanjing Medical University, Huai’an, Jiangsu, China; 2 Department of Radiation Oncology, The Affiliated Huai’an No. 1 People’s Hospital of Nanjing Medical University, Huai’an, Jiangsu, China; 3 Department of Pathology, The Affiliated Huai’an No. 1 People’s Hospital of Nanjing Medical University, Huai’an, Jiangsu, China; 4 Department of Thoracic Surgery, The Affiliated Huai’an No. 1 People’s Hospital of Nanjing Medical University, Huai’an, Jiangsu, China; 5 Department of Oncology, Nanjing Drum Tower Hospital, Affiliated Hospital of Medical School, School of Life Sciences, Nanjing University, Nanjing, China; 6 Department of Respiration, The Affiliated Huaian No. 1 People’s Hospital of Nanjing Medical University, Huai’an, Jiangsu, China

**Keywords:** IL-15, immunotherapy, malignant pleural effusion, *Mycobacterium* smegmatis, PD-L1, tumor microenvironment

## Abstract

**Background:**

Malignant pleural effusion (MPE) is a common and devastating complication of advanced malignancies, particularly lung cancer, with limited therapeutic options and poor prognosis. Immune checkpoint inhibitors and cytokine therapies have shown promise in solid tumors but are constrained by dose-dependent toxicity and limited efficacy in the pleural cavity. Engineered *Mycobacterium smegmatis* (*M. smegmatis*) co-expressing a PD-L1 inhibitor and interleukin-15 immunocytokine (Ms-PI15) represents a novel *in situ* vaccine platform capable of sustained local drug delivery with favorable biosafety profiles. Here, we investigated the therapeutic efficacy and underlying mechanisms of intrapleural Ms-PI15 administration in a murine MPE model.

**Methods:**

A syngeneic MPE mouse model was established by intrapleural inoculation of luciferase-expressing Lewis lung carcinoma (LLC-Luc) cells into C57BL/6 mice. Mice were treated with intrapleural injections of PBS, Ms-vector (empty vector control), or Ms-PI15. Tumor burden was monitored by bioluminescence imaging. Pharmacokinetics and biodistribution of Ms-PI15 were evaluated using DIR fluorescence imaging and bacterial colony enumeration. Flow cytometry and immunohistochemistry were employed to characterize immune cell populations in the tumor microenvironment, spleen, and thoracic draining lymph nodes (TDLNs). Biosafety was assessed by histopathological examination and serum biochemistry.

**Results:**

Intrapleural administration of Ms-PI15 significantly suppressed MPE development and pleural tumor growth compared with PBS and Ms-vector controls. DIR fluorescence imaging and CFU enumeration demonstrated that Ms-PI15 accumulated predominantly in the pleural tumor microenvironment and TDLNs, peaking at 72 h post-administration, with minimal dissemination to distant organs. Mechanistically, Ms-PI15 remodeled the immune microenvironment by promoting maturation of CD80^+^CD86^+^ myeloid dendritic cells (mDCs), driving M1 polarization of tumor-associated macrophages (increased CD86^+^, decreased CD206^+^), and significantly enhancing infiltration of CD8 T cells and NK1.1^+^ NK cells in the pleural tumor, spleen, and TDLNs. No significant histopathological abnormalities or serum biochemical alterations were observed in major organs following Ms-PI15 treatment, confirming its favorable biosafety profile.

**Conclusion:**

Intrapleural administration of the engineered *M. smegmatis* Ms-PI15 effectively inhibits MPE progression by remodeling the pleural immune microenvironment toward an antitumor phenotype, while maintaining excellent biosafety. These findings provide a preclinical foundation for the development of intracavitary bacterial immunotherapy as a novel treatment strategy for malignant pleural effusion.

## 1 Introduction

Malignant pleural effusion (MPE) is a frequent and severe complication of advanced malignancies, affecting approximately 15% of patients with cancer during the disease course (Feller-Kopman and Light, 2018; Cengiz et al., 2026; Porcel, 2026). Lung cancer, breast cancer, and lymphoma are the most common primary tumors associated with MPE (Heffner and Klein, 2008; Wright et al., 2026). The presence of MPE indicates advanced disease stage and is associated with significantly reduced survival, with a median survival of only 4–6 months following diagnosis (Hassan et al., 2021; Zhang X. et al., 2021). The pathogenesis of MPE involves a complex interplay between tumor cells and the pleural microenvironment, characterized by increased vascular permeability, impaired lymphatic drainage, and profound immunosuppression within the pleural cavity (Song et al., 2021; Ge et al., 2024). Despite its clinical significance, therapeutic options for MPE remain limited. Current standard treatments include therapeutic thoracentesis, chemical pleurodesis, and indwelling pleural catheters, which primarily address symptom relief rather than targeting the underlying tumor biology (Hughes and Carmichael, 2022; Shaw et al., 2026). Systemic chemotherapy and targeted therapies have shown modest efficacy, and the unique immunosuppressive milieu of the pleural cavity poses substantial challenges for conventional immunotherapies (Bondi et al., 2026).

The pleural immune microenvironment in MPE is characterized by a predominance of immunosuppressive cell populations, including M2-polarized tumor-associated macrophages (TAMs), regulatory T cells (Tregs), and myeloid-derived suppressor cells (MDSCs), alongside impaired dendritic cell (DC) maturation and reduced cytotoxic T lymphocyte (CTL) infiltration (Kaczmarek and Sikora, 2012; Awad et al., 2016; Fukui et al., 2025). This “cold” immune landscape limits the efficacy of immune checkpoint inhibitors (ICIs) that have revolutionized treatment for many solid tumors (Sharma and Allison, 2015). Furthermore, intrapleural administration of ICIs or cytokines faces pharmacokinetic challenges, including rapid clearance from the pleural space and dose-limiting systemic toxicity (Bertoglio et al., 2018; Nowak et al., 2021). Therefore, there is an urgent need for novel therapeutic strategies that can sustainably modulate the pleural immune microenvironment while maintaining a favorable safety profile.

Engineered bacteria-based *in situ* vaccine platforms have emerged as a promising approach for cancer immunotherapy (Zheng et al., 2017; Chowdhury et al., 2019; Gurbatri et al., 2020). *Mycobacterium smegmatis* (*Mycobacterium smegmatis*), a non-pathogenic mycobacterial species, possesses inherent immunostimulatory properties as a potent adjuvant without endotoxin production, making it an attractive vehicle for therapeutic protein delivery (Lamensans et al., 1975; Decroix et al., 1984; Jeong et al., 2021). Compared with the FDA-approved *Mycobacterium bovis Bacillus Calmette-Guérin* (*BCG*), *M. smegmatis* exhibits faster growth kinetics, more efficient genetic manipulation, and greater susceptibility to phagolysosomal degradation, which facilitates enhanced antigen release and cross-presentation by DCs (Young et al., 2004; Rich et al., 2012; Wang et al., 2025). We have previously engineered *M. smegmatis* to co-express a single-chain variable fragment (scFv) targeting PD-L1 and an IL-15 cytokine complex containing the IL-15 receptor alpha (IL-15Rα) sushi domain (Ms-PI15) (Mei et al., 2025). This bifunctional fusion protein simultaneously blocks the PD-L1 immune checkpoint pathway and provides sustained IL-15 signaling, which promotes NK cell activation and CD8^+^ T cell proliferation. We demonstrated that Ms-PI15 effectively overcomes resistance to PD-L1 blockade, induces DC maturation, promotes M1 macrophage polarization, and elicits robust antitumor immunity in multiple subcutaneous tumor models (Mei et al., 2025).

Building upon these findings, we hypothesized that intrapleural administration of Ms-PI15 could effectively remodel the immunosuppressive pleural microenvironment and suppress MPE progression. In this study, we established a syngeneic MPE mouse model using luciferase-expressing Lewis lung carcinoma (LLC-Luc) cells and evaluated the therapeutic efficacy, pharmacokinetics, immune-modulatory mechanisms, and biosafety of intrapleural Ms-PI15 administration. Our results demonstrate that Ms-PI15 significantly inhibits MPE development and pleural tumor growth through comprehensive remodeling of the pleural immune microenvironment, while exhibiting excellent biosafety with no discernible organ toxicity. These findings establish intrapleural Ms-PI15 as a promising therapeutic strategy for malignant pleural effusion.

## 2 Materials and methods

### 2.1 Bacterial strains and cell lines

*Mycobacterium smegmatis* mc^2^155, *Escherichia coli* BL21, and the pMV261 mycobacterial shuttle vector were maintained in our laboratory. Engineered *M. smegmatis* strains (Ms-PI15 and Ms-vector) were constructed as previously described (Mei et al., 2025). Briefly, the complementary DNA encoding the PD-L1 scFv–IL-15 fusion protein was cloned into the pMV261 vector under the control of the constitutively expressed mycobacterial heat shock protein 60 (Hsp60) promoter and the α-antigen signal peptide of BCG. Plasmids were introduced into *M. smegmatis* by electroporation at 2.5 kV, 25 μF, and 1,000 Ω using a 2 mm cuvette (Bio-Rad, Richmond, CA, United States). Transformants were selected on 7H10 agar containing kanamycin (50 μg/mL). *M. smegmatis* was grown in Middlebrook 7H9 broth (BD Biosciences, Milpitas, United States) supplemented with 10% oleic acid-albumin-dextrose-catalase (OADC, BD Biosciences), 0.2% (v/v) glycerol, 0.05% (v/v) Tween 80, and kanamycin (50 μg/mL) at 37 °C with shaking at 220 rpm. Enumeration of colony-forming units (CFU) was performed by serial dilution and plating on 7H10 agar.

The Lewis lung carcinoma cell line expressing luciferase (LLC-Luc) was generated by lentiviral transfection of LLC cells with a luciferase-expressing lentivirus (GeneChem, Shanghai, China). LLC cells were obtained from the Cell Bank of Shanghai Institute of Biochemistry and Cell Biology (Shanghai, China) and maintained in Roswell Park Memorial Institute (RPMI) 1640 medium (Gibco, Thermo Fisher Scientific, Grand Island, United States) supplemented with 10% fetal bovine serum (FBS, Gibco), 100 U/mL penicillin, and 100 μg/mL streptomycin at 37 °C in a humidified 5% CO_2_ incubator.

### 2.2 Animals

Male C57BL/6 mice aged 5–6 weeks were purchased from Shanghai Sippr-BK Laboratory Animal Co., Ltd. (Shanghai, China) and housed in the specific pathogen-free (SPF) Laboratory Animal Center. All experimental protocols were approved by the Laboratory Animal Care and Use Committee of the Affiliated Nanjing Drum Tower Hospital of Nanjing University Medical School. After intrapleural inoculation of LLC-Luc cells, tumor-bearing mice were randomly assigned to treatment groups. In the dose-finding experiment, mice received intrapleural PBS, Ms-PI15 at 1 × 10^7^ CFU, or Ms-PI15 at 1 × 10^8^ CFU, with three mice per group. In the therapeutic efficacy experiment, mice received intrapleural PBS, Ms-vector, or Ms-PI15, with four mice per group for tumor burden and malignant pleural effusion assessment. An independent cohort was used for survival analysis, in which mice were assigned to the same three treatment groups, with five mice per group. All anesthesia and euthanasia procedures were performed in accordance with the approved institutional animal care and use protocol.

### 2.3 Establishment of the MPE mouse model and treatment

For MPE model establishment, LLC-Luc cells were harvested, washed three times in PBS, and resuspended at 2 × 10^6^ cells/mL in PBS. C57BL/6 mice were anesthetized with inhaled isoflurane (3%–4% for induction and 1.5%–2% for maintenance), and 100 µL of cell suspension (2 × 10^5^ cells per mouse) was injected into the left pleural cavity. Mice were randomized into treatment groups 5 days after tumor inoculation.

For the dose-optimization study, mice received intrapleural injections of PBS, 10^7^ CFU Ms-PI15, or 10^8^ CFU Ms-PI15 (100 µL per injection) on Days 5, 10, and 15 post-tumor inoculation. For subsequent experiments, mice were treated with PBS, Ms-vector (10^8^ CFU), or Ms-PI15 (10^8^ CFU) on the same schedule. All intrapleural injections were performed under isoflurane anesthesia using the same anesthesia protocol described above. Bacteria were cultured to the logarithmic growth phase in 7H9 broth, harvested by centrifugation, washed three times with ice-cold PBS, and resuspended to the desired concentration.

### 2.4 *In vivo* bioluminescence imaging

Tumor burden was monitored using the IVIS Spectrum *In Vivo* Imaging System (PerkinElmer, Waltham, United States). Mice were anesthetized with isoflurane and injected intraperitoneally with D-luciferin potassium salt (150 mg/kg, PerkinElmer). Bioluminescence images were acquired 10 min after luciferin injection. Total radiance efficiency (photons/s/cm^2^/sr) in the region of interest (ROI) over the chest area was quantified using Living Image software (PerkinElmer).

### 2.5 DIR fluorescence imaging and biodistribution analysis

For pharmacokinetic and biodistribution studies, Ms-PI15 was labeled with the near-infrared fluorescent dye DiR (Bridgen, Beijing, China) according to the manufacturer’s instructions. DiR-labeled Ms-PI15 (10^8^ CFU) was administered intrapleurally to LLC-bearing mice. *In vivo* fluorescence imaging was performed at 6, 24, 72, 168, and 336 h post-administration using the IVIS Spectrum system with appropriate excitation and emission filters for DiR. For *ex vivo* imaging, mice were euthanized at the indicated timepoints, and tumors, TDLNs, and major organs (heart, liver, spleen, lung, kidney) were harvested and imaged immediately.

### 2.6 Bacterial colonization assay

At designated timepoints (6, 24, 72, 168, and 336 h) post-intrapleural administration of Ms-PI15, mice were euthanized and tumor, TDLN, heart, liver, spleen, lung, kidney, and blood samples were collected. Tissues were weighed and homogenized in sterile PBS at 4 °C using a tissue homogenizer. Homogenates were serially diluted and plated on 7H10 agar plates. Plates were incubated at 37 °C for 3–6 days, and bacterial colonies were counted. Results were expressed as CFU per gram of tissue (or CFU per mL for blood).

### 2.7 Flow cytometry analysis

Tumor tissues, TDLNs, and spleens were harvested at the experimental endpoint. Single-cell suspensions from spleens and TDLNs were prepared by mechanical trituration through a 70 μm cell strainer. For tumor-infiltrating lymphocyte isolation, tumor tissues were minced and digested in RPMI 1640 medium containing collagenase type IV (1 mg/mL, Sigma-Aldrich, St. Louis, United States) at 37 °C for up to 2 h with gentle agitation. Digested tissues were filtered through a 70 µm strainer to obtain single-cell suspensions.

Cells (1 × 10^6^ per sample) were stained with fluorochrome-conjugated antibodies for 20 min at room temperature in the dark. The following antibodies were used: FITC-CD11c (clone N418, catalog #117306), APC-CD80 (clone 16-10A1, #104714), PE-CD86 (clone GL-1, #105008), PerCP/Cyanine5.5-CD8a (clone 53-6.7, #100734), FITC-CD3 (clone 145-2C11, #152304), PE-CD4 (clone GK1.5, #100408), APC-CD8a (clone 53-6.7, #100712), PE-NK-1.1 (clone S17016D, #156504), PE/Cyanine7-CD69 (clone H1.2F3, #104512), PE-CD206 (clone C068C2, #141706), APC-Cyanine7-F4/80 (clone BM8, #123112), APC-CD11b (clone M1/70, #101212), and Fixable Viability Dye BV510 (#423121). All flow cytometry antibodies were purchased from BioLegend (San Diego, United States). Samples were analyzed using a BD Accuri C6 flow cytometer (BD Biosciences, United States), and data were processed with FlowJo software (Tree Star, Ashland, United States).

For flow-cytometric analysis, debris was first excluded based on FSC-A/SSC-A profiles, followed by doublet exclusion using FSC-A/FSC-H. Dead cells were excluded using fixable viability dye BV510, and subsequent analyses were performed by gating on live single cells. Immune-cell subsets were then identified using the indicated lineage and functional markers.

### 2.8 Immunohistochemistry

Tumor tissues were fixed in 4% paraformaldehyde, embedded in paraffin, and sectioned at 4 µm thickness. After deparaffinization and antigen retrieval, sections were incubated with anti-mouse CD8α primary antibody (Abcam, Cambridge, United Kingdom) at 4 °C overnight, followed by incubation with horseradish peroxidase (HRP)-conjugated secondary antibody (Beyotime, Shanghai, China) for 1 h at room temperature. Immunoreactivity was visualized using 3,3′-diaminobenzidine (DAB) substrate, and sections were counterstained with hematoxylin. Slides were scanned using a Zeiss Axioscan.Z1 scanner with a 20× objective, and analysis was performed in ImageJ software (National Institutes of Health, Bethesda, United States).

### 2.9 Histopathological examination

Major organs (heart, liver, spleen, lung, and kidney) were harvested, fixed in 4% paraformaldehyde, embedded in paraffin, sectioned, and stained with hematoxylin and eosin (H&E). Slides were scanned on a Zeiss Axioscan.Z1 with a 20× objective and analyzed using ImageJ software.

### 2.10 Serum biochemistry analysis

At the experimental endpoint, mice were deeply anesthetized with inhaled isoflurane (3%–4% for induction, followed by 1.5%–2% for maintenance). Blood samples were collected by terminal cardiac puncture under deep anesthesia. Immediately after blood collection, the mice were euthanized by cervical dislocation while still under deep anesthesia. Blood samples were centrifuged at 3,000 × g for 15 min to obtain serum. Serum levels of alanine aminotransferase (ALT), aspartate aminotransferase (AST), alkaline phosphatase (AKP), creatinine (CREA), and urea were measured using an automatic biochemical analyzer (Hitachi, Tokyo, Japan) according to standard protocols.

### 2.11 Statistical analysis

All statistical analyses were performed using GraphPad Prism software (version 9.0, GraphPad Software, San Diego, United States). Data are presented as the mean ± standard error of the mean (s.e.m.). Comparisons between two groups were performed using two-tailed Student’s t-test. Comparisons among multiple groups were performed using one-way analysis of variance (ANOVA) with Tukey’s *post hoc* test. Longitudinal data (tumor burden over time, body weight over time) were analyzed using two-way ANOVA with Tukey’s multiple comparisons test. A *p*-value <0.05 was considered statistically significant. Significance levels are denoted as follows: *p < 0.05, **p < 0.01, ***p < 0.001, ****p < 0.0001, and ns (not significant) for *p* > 0.05.

## 3 Results

### 3.1 Establishment of the MPE mouse model and determination of the optimal dosage of intrapleural Ms-PI15

To evaluate the therapeutic efficacy of Ms-PI15 in the context of malignant pleural effusion, we first established a syngeneic MPE mouse model using C57BL/6 mice intrapleurally inoculated with luciferase-expressing Lewis lung carcinoma cells (LLC-Luc, 2 × 10^5^ cells per mouse). Pleural effusion smears obtained on Day 15 post-tumor inoculation confirmed the presence of tumor cells in the pleural fluid under optical microscopy, validating successful establishment of the MPE model (Figure 1B).

**FIGURE 1:**
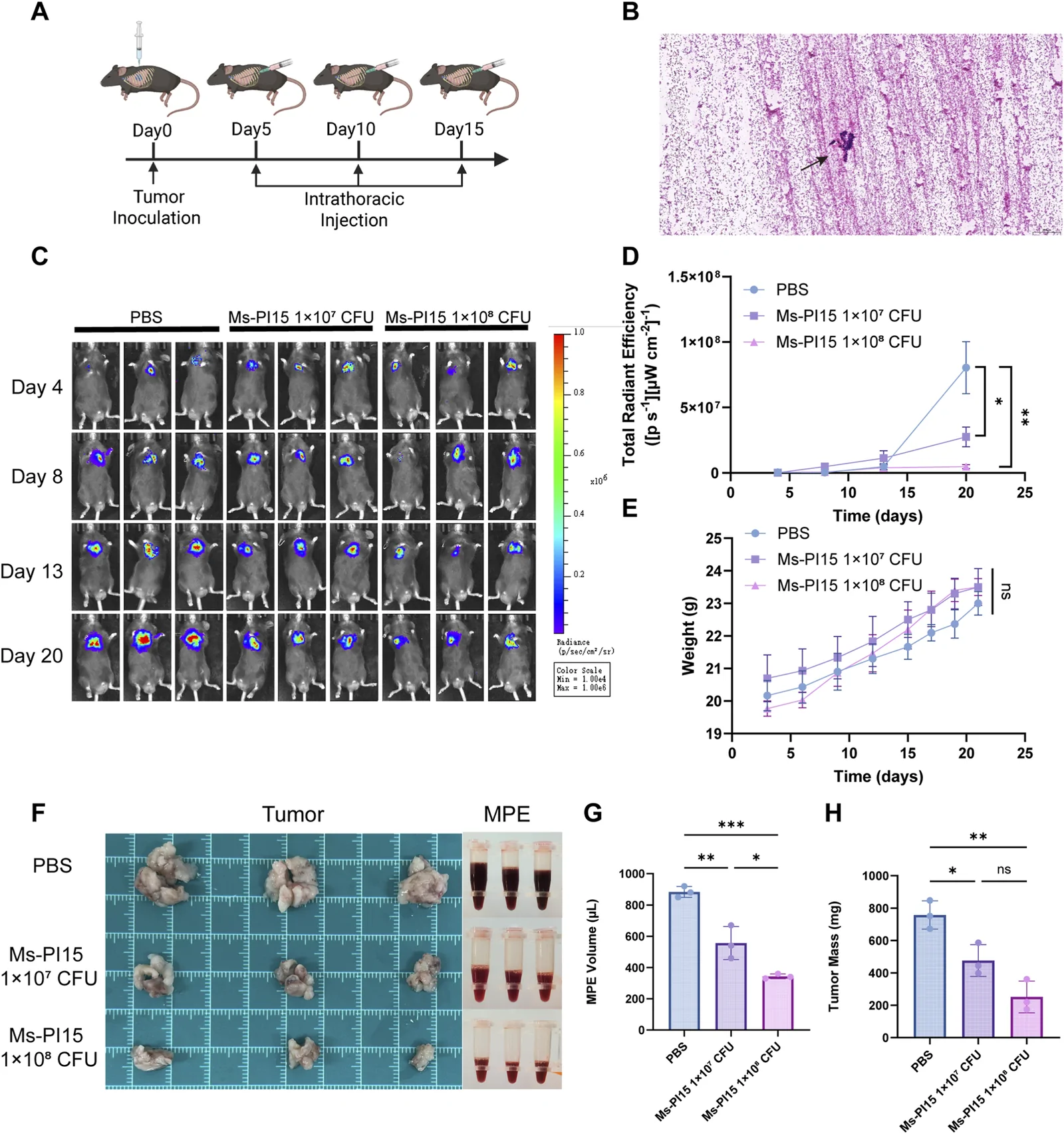
Establishment of MPE animal models and the optimal dosage of intrapleural administered Ms-PI15. **(A)** Scheme showing the design of the animal experiment. C57BL/6 mice were intrapleurally inoculated with LLC-Luc cells (2 × 10^5^ per mouse) and randomized into three groups with treated intrapleurally with Ms-PI15. Created with BioRender.com. **(B)** Pleural effusion smears obtained from LLC-Luc-bearing mouse on Day 15 of tumor inoculation revealed tumor cells under optical microscopy. The scale bars were 50 μm. **(C)** Intrapleural tumor burden monitored with IVIS of mice administered with PBS, 10^7^ CFU Ms-PI15, 10^8^ CFU Ms-PI15 on Days 4, 8, 13 and 20. **(D)** Bioluminescence intensity in region of interest (ROI) within the chest area (n = 3). **(E)** Average weight of LLC MPE mice in different groups over 21 days (n = 3). **(F)** Representative photographs of MPEs and pleural tumors harvested from mice in different groups on Day 21. **(G,H)** MPE volume **(G)** and pleural tumor weight **(H)** on Day 21 in the LLC-Luc MPE mice (n = 3). For the experiments in **(D,E)** data were the mean ± s.e.m. *p*-values were determined by two-way ANOVA with Tukey’s multiple comparisons test. For the experiments in **(G,H)** data were the mean ± s.e.m. *p*-values were determined by one-way ANOVA with Tukey’s *post hoc* test. * represented *p* < 0.05, ** represented *p* < 0.01, *** represented *p* < 0.001, and ns represented *p* > 0.05.

Mice were randomized into three treatment groups receiving intrapleural injections of PBS (vehicle control), 10^7^ CFU Ms-PI15, or 10^8^ CFU Ms-PI15 on Days 5, 10, and 15 post-tumor inoculation (Figure 1A). Intrapleural tumor burden was monitored longitudinally using bioluminescence imaging (IVIS) on Days 4, 8, 13, and 20. Mice treated with 10^8^ CFU Ms-PI15 exhibited significantly reduced bioluminescence signal in the region of interest (ROI) within the chest area compared with both the PBS and 10^7^ CFU Ms-PI15 groups, indicating dose-dependent suppression of pleural tumor growth (Figures 1C,D). No significant differences in body weight were observed among the treatment groups throughout the 21-day observation period, indicating good tolerability and favorable biosafety of Ms-PI15 administration (Figure 1E).

On Day 21, mice were euthanized and pleural effusion volume and tumor mass were quantified. Representative photographs demonstrated markedly reduced pleural effusion and smaller pleural tumors in the 10^8^ CFU Ms-PI15 group compared with controls (Figure 1F). Quantitative analysis confirmed that both MPE volume (Figure 1G) and pleural tumor weight (Figure 1H) were significantly lower in the 10^8^ CFU Ms-PI15 group than in the PBS and 10^7^ CFU Ms-PI15 groups. These results established 10^8^ CFU as the optimal dosage for subsequent experiments and validated the MPE mouse model for evaluating the therapeutic efficacy of intrapleural Ms-PI15.

### 3.2 Intrapleural administration of Ms-PI15 effectively inhibits MPE development and pleural tumor growth

Having established the optimal dosage, we next evaluated the therapeutic efficacy of Ms-PI15 in comparison with an empty vector control (Ms-vector). C57BL/6 mice were intrapleurally inoculated with LLC-Luc cells (2 × 10^5^ per mouse) and subsequently treated with intrathoracic injections of PBS, Ms-vector, or Ms-PI15 (10^8^ CFU) on Days 5, 10, and 15 (Figure 2A). Intrapleural tumor burden was monitored by IVIS bioluminescence imaging on Days 4, 8, 13, and 20. Representative images revealed progressive tumor growth in both the PBS and Ms-vector groups, whereas the Ms-PI15 group exhibited markedly attenuated bioluminescence signals throughout the observation period, indicating effective suppression of pleural tumor proliferation (Figure 2B). Quantification of total radiance efficiency in the chest ROI confirmed that Ms-PI15 treatment resulted in significantly lower tumor burden compared with both control groups from Day 13 onward (Figure 2C).

**FIGURE 2:**
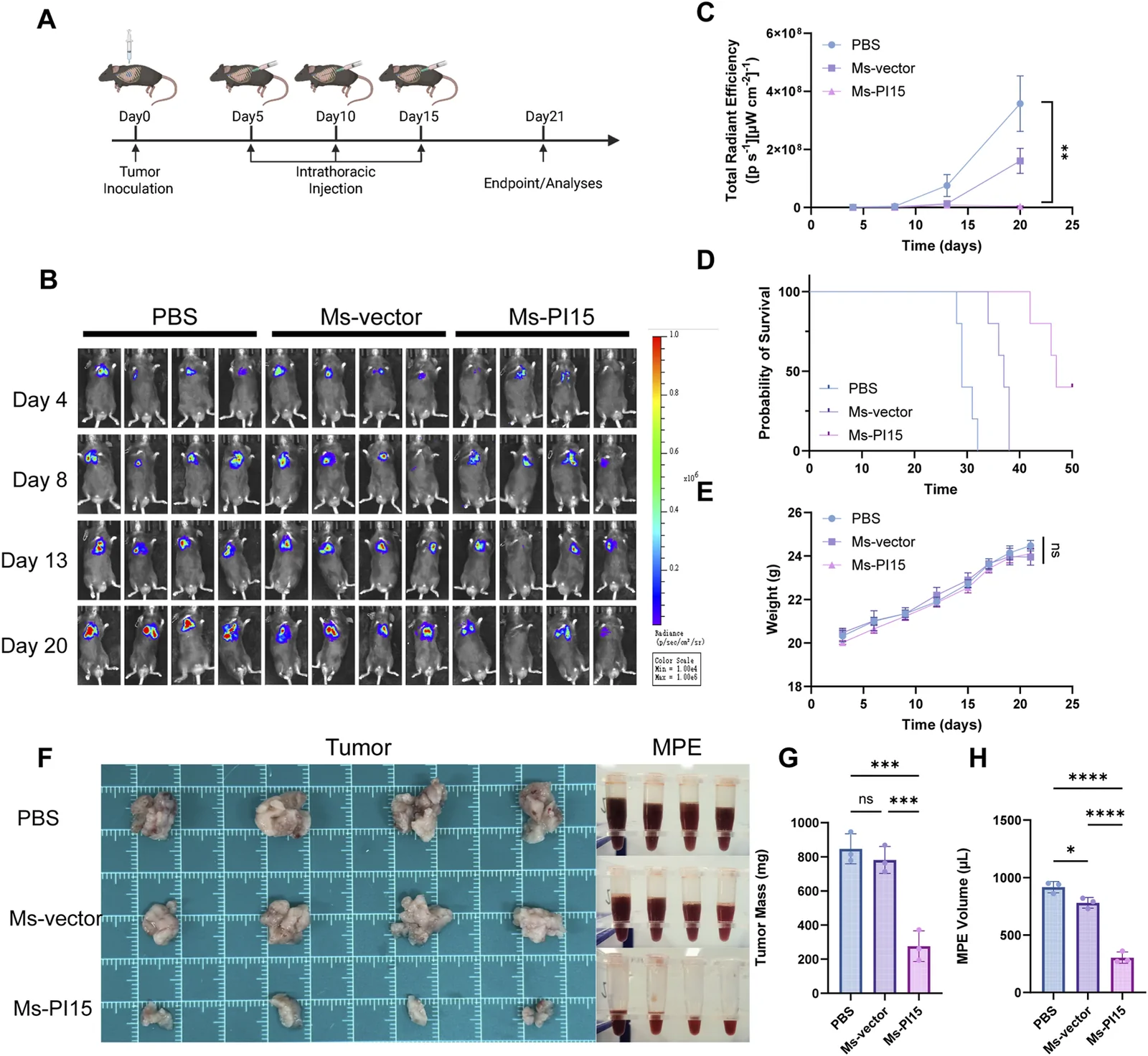
Intrapleural administration of Ms-PI15 effectively inhibited the development of MPE and pleural tumors in the LLC lung cancer model. **(A)** Schematic showing the design of the animal experiment. C57BL/6 mice were intrapleurally inoculated with LLC-Luc cells (2 × 10^5^ per mouse) and subsequently treated with intrathoracic injections of PBS, Ms-vector, or Ms-PI15 on Days 5, 10, and 15. Created with BioRender.com. **(B)** Intrapleural tumor burden monitored with IVIS bioluminescence imaging of mice administered with PBS, Ms-vector, or Ms-PI15 on Days 4, 8, 13, and 20. Representative images from each group are shown. **(C)** Total radiance efficiency in ROI within the chest area over time (n = 4 per group). **(D)** Kaplan-Meier survival curves of mice in different treatment groups (n = 5). Median survival times were 29 days for PBS, 37 days for Ms-vector, and 47 days for Ms-PI15. Mice alive at Day 50 in the Ms-PI15 group were censored. **(E)** Average body weight of MPE mice in different treatment groups over 21 days (n = 4). **(F)** Representative photographs of pleural tumors and MPE fluid collected from mice in different groups at the endpoint. **(G,H)** Tumor mass **(G)** and MPE volume **(H)** in the LLC-Luc MPE mice (n = 4 per group). For the experiments in **(C,E)** data were the mean ± s.e.m. p-values were determined by two-way ANOVA with Tukey’s multiple comparisons test. For the experiment in **(D)** survival differences were analyzed using the log-rank Mantel-Cox test. The overall log-rank test showed a significant difference among groups, *p* < 0.0001. For the experiments in **(G,H)** data were the mean ± s.e.m. p-values were determined by one-way ANOVA with Tukey’s *post hoc* test. * represented *p* < 0.05, ** represented *p* < 0.01, *** represented *p* < 0.001, **** represented *p* < 0.0001, and ns represented *p* > 0.05.

Survival analysis was performed to evaluate the impact of Ms-PI15 treatment on overall survival. Kaplan-Meier survival curves demonstrated that intrapleural Ms-PI15 administration significantly prolonged survival compared with both the PBS and Ms-vector groups. The median survival times were 29, 37, and 47 days for the PBS, Ms-vector, and Ms-PI15 groups, respectively. These results indicate that suppression of pleural tumor growth translated into a meaningful survival benefit (Figure 2D).

Body weight was monitored as an indicator of overall health status. Mice in all three groups maintained relatively stable body weight throughout the 21-day experimental period, with no significant differences among groups, suggesting that intrapleural Ms-PI15 administration was well tolerated (Figure 2E). At the experimental endpoint (Day 21), representative photographs of harvested pleural tumors and MPE fluid demonstrated visibly smaller tumor masses and reduced effusion volume in the Ms-PI15 group compared with PBS and Ms-vector controls (Figure 2F). Quantitative analysis confirmed that both tumor mass (Figure 2G) and MPE volume (Figure 2H) were significantly reduced in the Ms-PI15 group, with highly significant differences (p < 0.001 for tumor mass; p < 0.0001 for MPE volume) compared with controls. Notably, no significant differences were observed between the PBS and Ms-vector groups, indicating that the antitumor effect was specifically attributable to the PD-L1/IL-15 fusion protein expressed by Ms-PI15 rather than to the bacterial vector alone.

### 3.3 Pharmacokinetics and biodistribution of Ms-PI15 in the MPE mouse model

To understand the spatial and temporal dynamics of Ms-PI15 following intrapleural administration, we performed pharmacokinetic and biodistribution studies using DIR-labeled bacteria. DIR-labeled Ms-PI15 was administered intrapleurally to LLC-bearing mice, and fluorescence imaging was performed at 6, 24, 72, 168, and 336 h post-treatment. *In vivo* imaging revealed that Ms-PI15 accumulated predominantly in the pleural tumor region, with fluorescence intensity peaking at 72 h and subsequently declining over time (Figure 3A, top row; Figure 3B). *Ex vivo* imaging of resected thoracic tissues confirmed the persistent presence of Ms-PI15 at the tumor site and demonstrated detectable fluorescence in the thoracic draining lymph nodes (TDLNs), suggesting bacterial migration to or immune cell-mediated transport to the draining lymphatics (Figure 3A, bottom row; Figure 3C). Importantly, minimal fluorescence signals were detected in distant organs, indicating favorable localization and limited systemic dissemination following intrapleural administration.

**FIGURE 3:**
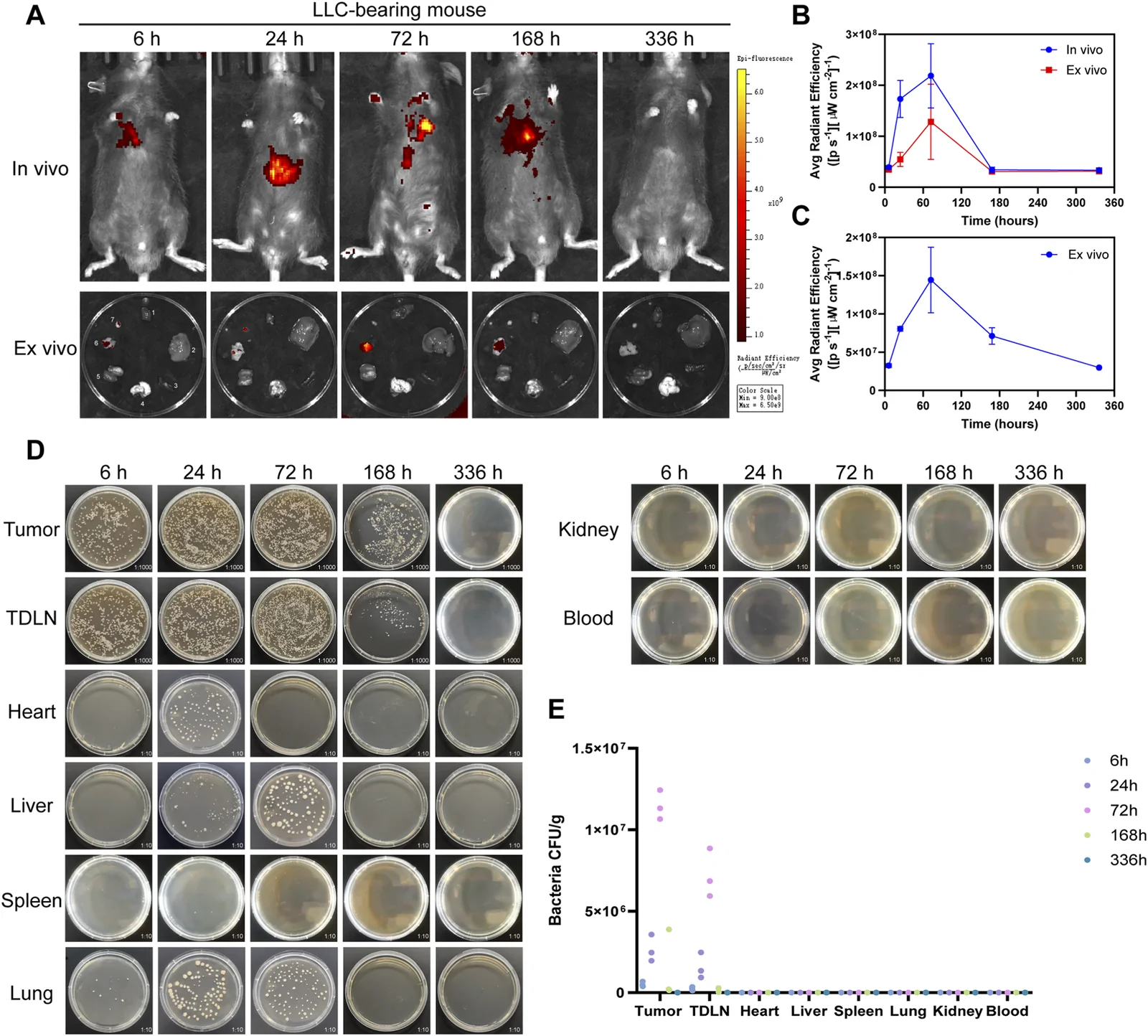
Pharmacokinetics and biodistribution of Ms-PI15 in the MPE mouse model. **(A)** Representative in vivo (top row) and ex vivo (bottom row) DIR fluorescence imaging of LLC-bearing mice following intrapleural administration of DiR-labeled Ms-PI15. Images were acquired at 6, 24, 72, 168, and 336 h post-treatment. In the ex vivo images, the numbered tissues are indicated as follows: 1, heart; 2, liver; 3, spleen; 4, lung; 5, kidney; 6, tumor; and 7, lymph nodes. **(B)** Quantification of average radiance efficiency in the in vivo and ex vivo imaging over time (n = 3). **(C)** Quantification of average radiance efficiency in thoracic draining lymph node (TDLN) ex vivo imaging over time (n = 3). **(D)** Bacterial colony counts on agar plates from various tissues, including tumor, TDLN, heart, liver, spleen, lung, kidney, and blood, collected at 6, 24, 72, 168, and 336 h post-treatment. **(E)** Quantification of bacterial CFU per gram of tissue (or per mL for blood) in each organ at different timepoints. For the experiments in **(B,C)** data are shown as the mean ± s.d. of n = 3 biologically independent samples.

To complement the fluorescence imaging data, we quantified viable bacterial colonization across multiple tissues. Agar plate colony counts were performed on homogenized samples from tumor, TDLN, heart, liver, spleen, lung, kidney, and blood collected at the same timepoints (6, 24, 72, 168, and 336 h). Colony-forming units (CFU) were highest in the tumor tissue at 72 h, consistent with the fluorescence imaging results, and gradually decreased thereafter (Figures 3D,E). Viable bacteria were also detected in the TDLN, corroborating the imaging findings. In contrast, bacterial colonization in heart, liver, spleen, lung, kidney, and blood was negligible or below the detection limit at all timepoints examined (Figures 3D,E). These data collectively demonstrate that intrapleural Ms-PI15 preferentially localizes to the pleural tumor microenvironment and draining lymph nodes, with limited systemic distribution, supporting its favorable safety profile for intracavitary administration.

### 3.4 M-PI15 remodels the tumor immune microenvironment in the MPE model

The pleural immune microenvironment plays a critical role in MPE pathogenesis and response to therapy. To elucidate the immunological mechanisms underlying the antitumor efficacy of Ms-PI15, we performed comprehensive flow cytometric analysis of immune cell populations in the pleural tumor, spleen, and TDLNs at the experimental endpoint.

We first examined the maturation status of dendritic cells and macrophage polarization in the tumor microenvironment. Ms-PI15 treatment significantly increased the proportion of CD80^+^CD86^+^ myeloid dendritic cells. (mDCs) among CD11c^+^ cells in the pleural tumor compared with PBS and Ms-vector controls (Figure 4A), indicating enhanced DC maturation and antigen-presenting capacity. Regarding macrophage polarization, Ms-PI15 treatment significantly reduced the percentage of CD206^+^ M2-like tumor-associated macrophages (TAMs) among CD11b^+^F4/80^+^ cells (Figure 4B) while simultaneously increasing the proportion of CD86^+^ M1-like macrophages (Figure 4C). These results demonstrate that Ms-PI15 promotes a shift from immunosuppressive M2 to antitumor M1 macrophage polarization within the pleural tumor microenvironment.

**FIGURE 4:**
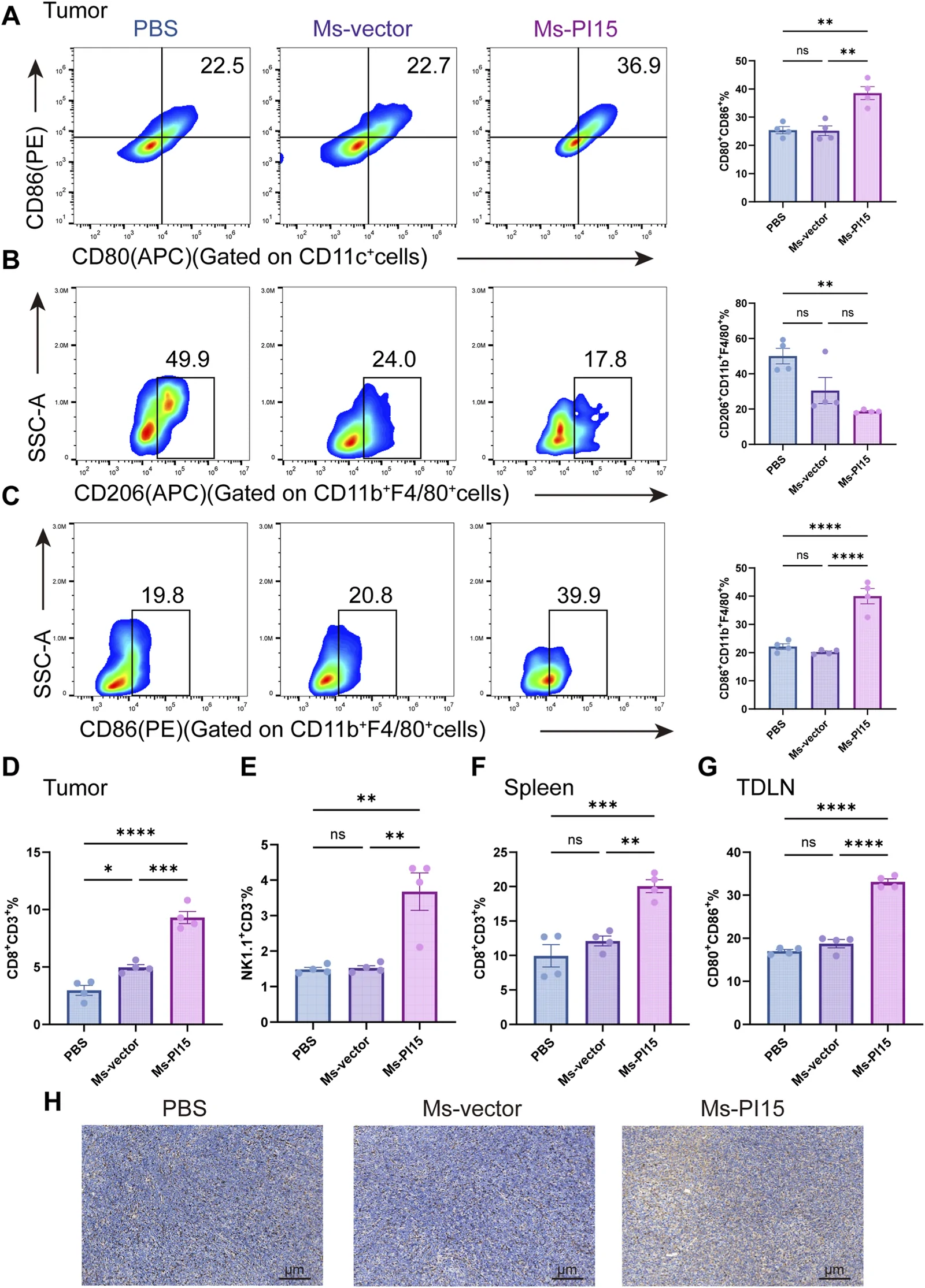
Ms-PI15 remodels the tumor immune microenvironment in the MPE mouse model. **(A–C)** Flow cytometric analysis of tumor-infiltrating immune cells. **(A)** Representative flow plots and quantification of CD80+CD86+ mDCs gated on tumor tissue from PBS, Ms-vector, and Ms-PI15 groups. **(B)** Representative flow plots and quantification of CD206+ cells among CD11b+F4/80+ cells (M2-like tumor-associated macrophages). **(C)** Representative flow plots and quantification of CD86+ cells among CD11b+F4/80+ cells (M1-like macrophages). **(D)** Percentage of CD8+ T cells in tumor tissue across different treatment groups. **(E)** Percentage of NK1.1+CD3– NK cells in tumor tissue. **(F)** Percentage of CD8+ T cells in the spleen. **(G)** Percentage of CD80+CD86+ mDCs in TDLN. **(H)** Representative immunohistochemistry images of CD8+ T cells in tumor tissue from mice in different groups. Scale bars, 100 μm. For the experiments in **(A–G)** data were the mean ± s.e.m. (n = 4). p-values were determined by one-way ANOVA with Tukey's post hoc test. * represented p < 0.05, ** represented p < 0.01, *** represented p < 0.001, **** represented p < 0.0001, and ns represented p > 0.05.

We next evaluated cytotoxic lymphocyte infiltration. Ms-PI15 treatment resulted in a significant increase in the percentage of CD8^+^ T cells in the tumor tissue (Figure 4D), accompanied by a significant elevation of NK1.1^+^CD3^−^ natural killer (NK) cells (Figure 4E), indicating robust recruitment and/or expansion of cytotoxic effector cells. Importantly, the immunomodulatory effects of Ms-PI15 extended beyond the local tumor microenvironment. The percentage of CD8^+^ T cells was also significantly elevated in the spleen (Figure 4F), and the proportion of CD80^+^CD86^+^ mDCs was significantly increased in the TDLNs (Figure 4G), suggesting systemic immune activation following intrapleural Ms-PI15 administration.

To further validate the flow cytometry findings, we performed immunohistochemical staining of CD8^+^ T cells in tumor tissue sections. Representative images confirmed markedly increased CD8^+^ T cell infiltration in the Ms-PI15 group compared with PBS and Ms-vector controls (Figure 4H), providing spatial context for the enhanced cytotoxic T cell presence within the tumor microenvironment. Collectively, these results demonstrate that intrapleural Ms-PI15 comprehensively remodels the pleural immune microenvironment by promoting DC maturation, driving M1 macrophage polarization, and enhancing cytotoxic T cell and NK cell infiltration, thereby converting the immunosuppressive “cold” pleural microenvironment into an immunologically active “hot” tumor microenvironment.

### 3.5 Biosafety evaluation of Ms-PI15 treatment

Given that systemic administration of immune modulators can cause significant adverse effects, we conducted a comprehensive biosafety evaluation of intrapleural Ms-PI15 administration. Histopathological examination was performed on major organs, including heart, liver, spleen, lung, and kidney, harvested from mice in the PBS, Ms-vector, and Ms-PI15 groups. H&E-stained sections revealed normal tissue architecture and morphology across all organs in all three groups, with no evidence of inflammation, necrosis, or other pathological alterations attributable to Ms-PI15 treatment (Figure 5A).

**FIGURE 5:**
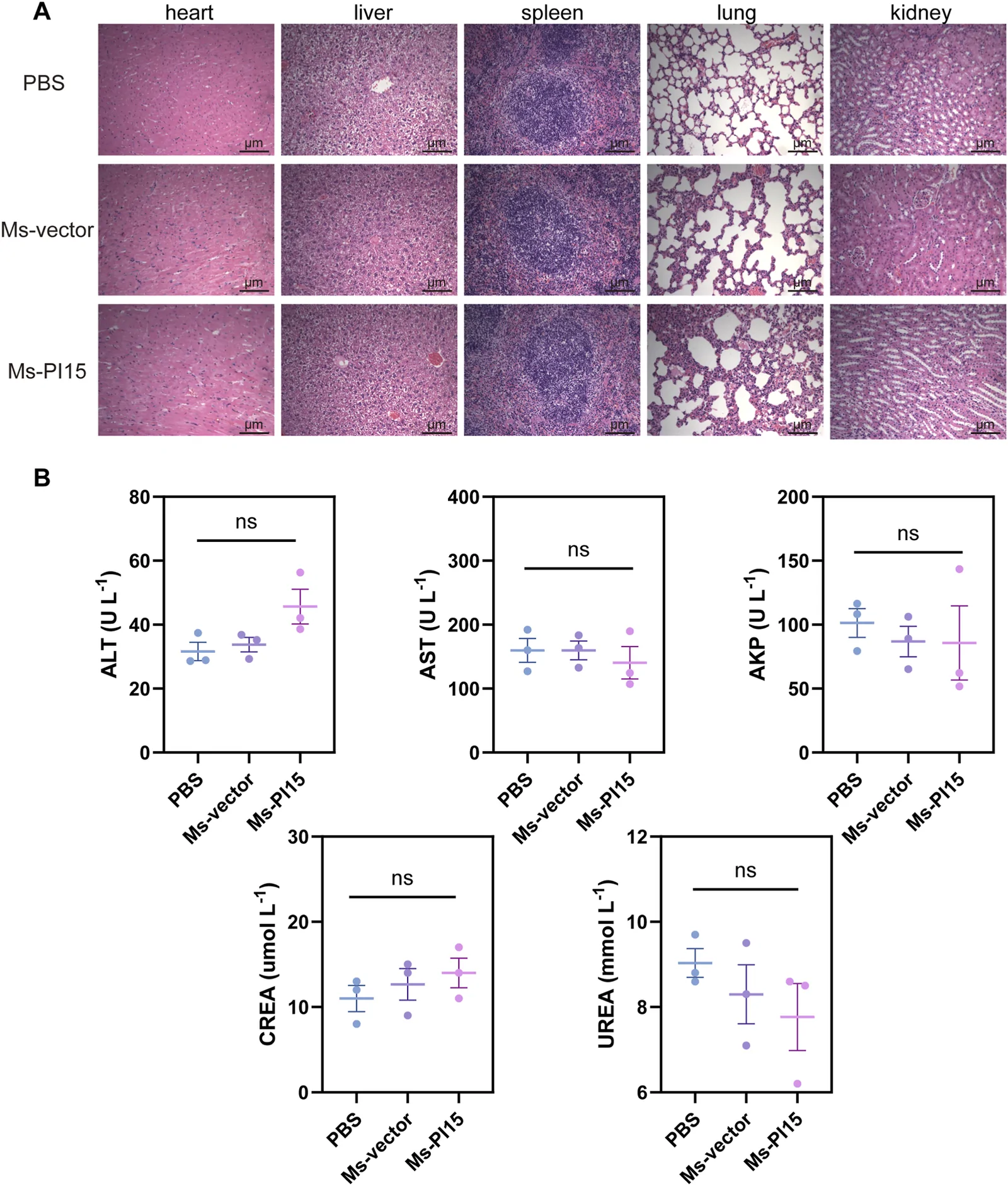
Biosafety evaluation of Ms-PI15 treatment. **(A)** Representative H&E-stained sections of major organs, including heart, liver, spleen, lung, and kidney, from mice in the PBS, Ms-vector, and Ms-PI15 groups. Scale bars, 100 μm. **(B)** Serum biochemical analysis of liver and kidney function indicators, including alanine aminotransferase (ALT), aspartate aminotransferase (AST), alkaline phosphatase (AKP), creatinine (CREA), and urea across different treatment groups. Data were the mean ± s.e.m. (n = 3). *p*-values were determined by one-way ANOVA with Tukey’s *post hoc* test. ns represented *p* > 0.05.

In parallel, serum biochemical analyses were performed to assess liver and kidney function. Levels of alanine aminotransferase (ALT), aspartate aminotransferase (AST), and alkaline phosphatase (AKP) — key indicators of hepatic function—showed no significant differences among the three treatment groups (Figure 5B). Similarly, creatinine (CREA) and urea levels, which reflect renal function, were comparable across groups (Figure 5B). These results confirm that intrapleural administration of Ms-PI15 does not induce detectable hepatotoxicity or nephrotoxicity. Together, the histopathological and biochemical data demonstrate the excellent biosafety profile of intrapleural Ms-PI15, supporting its potential for clinical translation.

## 4 Discussion

Malignant pleural effusion represents a significant clinical challenge in oncology, with limited therapeutic options and a dismal prognosis (Heffner and Klein, 2008; Feller-Kopman and Light, 2018; Hassan et al., 2021; Zhang X. et al., 2021; Cengiz et al., 2026; Wright et al., 2026). In this study, we demonstrate for the first time that intrapleural administration of the engineered *M. smegmatis* Ms-PI15 effectively suppresses MPE development and pleural tumor growth in a syngeneic mouse model through comprehensive remodeling of the pleural immune microenvironment.

The successful establishment of a reproducible MPE mouse model using LLC-Luc cells was critical for evaluating therapeutic interventions. Our dose-escalation study identified 10^8^ CFU as the optimal intrapleural dose of Ms-PI15, achieving significant tumor suppression without adverse effects on body weight. Notably, the empty vector control (Ms-vector) did not differ significantly from PBS in any efficacy endpoint, confirming that the antitumor activity is specifically attributable to the PD-L1/IL-15 fusion protein cargo rather than to the inherent immunostimulatory properties of the bacterial vector alone. Although *M. smegmatis* is intrinsically immunostimulatory, its nonspecific innate immune activation appeared insufficient to overcome the strongly immunosuppressive malignant pleural microenvironment and generate durable antitumor immunity. In contrast, Ms-PI15 integrates the adjuvant-like properties of the bacterial carrier with localized delivery of the therapeutic payload, thereby more effectively remodeling the pleural immune microenvironment and enhancing responsiveness to PD-L1 blockade. This finding is consistent with our previous study in subcutaneous tumor models.

The pharmacokinetic and biodistribution data revealed that intrapleurally administered Ms-PI15 exhibits favorable localization characteristics. DIR fluorescence imaging and CFU enumeration consistently demonstrated that bacteria accumulated predominantly at the pleural tumor site, with peak accumulation at 72 h post-administration. The detection of Ms-PI15 in the TDLN is particularly noteworthy, as it suggests that the bacteria or bacterial antigens are transported to the draining lymphatics, potentially facilitating antigen presentation and systemic immune activation. Importantly, negligible bacterial colonization was observed in distant organs (heart, liver, spleen, lung, kidney) and blood, indicating that intrapleural administration confines Ms-PI15 largely to the pleural compartment and regional lymphatics. This localized distribution pattern is advantageous compared with systemic administration, as it maximizes local therapeutic concentration while minimizing potential systemic adverse effects.

The immunological mechanisms underlying the efficacy of Ms-PI15 are multifaceted. The engineered bacteria simultaneously deliver two complementary immunomodulatory signals: PD-L1 blockade via the scFv moiety and IL-15-mediated immune stimulation via the cytokine moiety (Shi et al., 2023). This bifunctional design addresses two major barriers in the MPE microenvironment. First, the immunosuppressive pleural milieu, characterized by elevated PD-L1 expression on tumor cells and immune cells, inhibits effective T cell responses. By locally blocking PD-L1, Ms-PI15 removes this inhibitory signal and restores T cell effector function (Ohaegbulam et al., 2015; Baxi et al., 2018). Second, IL-15 is a potent cytokine that promotes the proliferation and activation of NK cells and memory CD8^+^ T cells (Zhang S. et al., 2021; Safaei et al., 2025). The sustained local production of IL-15 by Ms-PI15 provides continuous immune stimulation without the pharmacokinetic limitations of recombinant cytokine administration, which is characterized by a short half-life and dose-limiting toxicity (Guo et al., 2017; Fu et al., 2023).

Our flow cytometry analysis revealed that Ms-PI15 treatment induces a comprehensive shift in the immune cell composition of the pleural tumor microenvironment. The significant increase in CD80^+^CD86^+^ mDCs indicates enhanced antigen-presenting capacity, which is essential for initiating and sustaining antitumor T cell responses (Banchereau and Steinman, 1998). The concomitant decrease in CD206 M2-like TAMs and increase in CD86^+^ M1-like macrophages represents a favorable repolarization from an immunosuppressive to an immunostimulatory phenotype. TAMs are among the most abundant immune cell populations in MPE and play a central role in promoting tumor progression through secretion of pro-angiogenic factors, extracellular matrix remodeling, and suppression of cytotoxic lymphocytes (Fukui et al., 2025). The ability of Ms-PI15 to shift macrophage polarization toward the M1 phenotype is therefore a key contributor to its antitumor efficacy. The significant increase in CD8^+^ T cell and NK cell infiltration further demonstrates the successful conversion of the “cold” pleural microenvironment into a “hot” immunologically active state. The observation of elevated CD8^+^ T cells in the spleen and increased mDCs in the TDLN suggests that intrapleural Ms-PI15 administration induces systemic immune activation beyond the local pleural compartment, which may contribute to protection against metastatic dissemination.

The biosafety profile of intrapleural Ms-PI15 is excellent. H&E histopathology of major organs revealed no treatment-related abnormalities, and serum biochemical markers of liver and kidney function were unaltered across all treatment groups. These findings are consistent with the biodistribution data showing minimal systemic dissemination and are in agreement with our previous safety assessments in subcutaneous tumor models. The non-pathogenic nature of *M. smegmatis*, combined with its confinement to the pleural cavity following intrapleural administration, provides a wide therapeutic window.

Several limitations of this study should be acknowledged. First, the LLC MPE model, while well-established and reproducible, represents a single tumor histology. The efficacy of Ms-PI15 in MPE models derived from other primary tumors (e.g., breast cancer, mesothelioma) remains to be validated. Second, our study focused on the early-to-mid stage therapeutic effects; long-term survival studies and evaluation of immunological memory would provide additional insight into the durability of the response. Third, while we demonstrated comprehensive immune remodeling, the relative contributions of individual immune cell subsets to the overall therapeutic efficacy warrant further investigation through cell depletion studies. Fourth, the present study exclusively used male C57BL/6 mice, which does not allow for the evaluation of potential sex-specific differences in therapeutic efficacy or immune response. Future studies should include both male and female animals to determine whether the observed effects of Ms-PI15 are consistent across sexes, as sex hormones and genetic factors may influence immune responses and tumor biology. Fifth, the sample size was relatively small in some *in vivo* experiments, particularly those performed for mechanistic immune profiling. Although the observed effects were consistent across multiple readouts, larger animal cohorts will be required in future studies to confirm therapeutic efficacy, validate safety, and strengthen the translational relevance of this approach. Finally, translation to larger animal models and ultimately clinical trials will be necessary to establish the safety and efficacy of intrapleural Ms-PI15 in human MPE.

In conclusion, our study establishes intrapleural administration of the engineered *M. smegmatis* Ms-PI15 as a highly effective and safe therapeutic strategy for malignant pleural effusion. By simultaneously blocking PD-L1 and delivering IL-15 in a sustained, localized manner, Ms-PI15 comprehensively remodels the pleural immune microenvironment, promoting DC maturation, M1 macrophage polarization, and cytotoxic lymphocyte infiltration. These findings provide preliminary preclinical evidence supporting the further development of intracavitary bacterial immunotherapy as a potential treatment strategy for malignant pleural effusion.

## Data Availability

The original contributions presented in the study are included in the article/supplementary material, further inquiries can be directed to the corresponding author.
